# Giant left atrial thrombus in a patient with non-valvular atrial fibrillation

**DOI:** 10.1093/ehjcr/ytae606

**Published:** 2024-11-14

**Authors:** Yao Wang, Yan Liang

**Affiliations:** Emergency and Critical Care Center, Fuwai Hospital, National Center for Cardiovascular Diseases, Chinese Academy of Medical Sciences and Peking Union Medical College, Beilishi Road No. 167, Xicheng District, Beijing 100037, China; Emergency and Critical Care Center, Fuwai Hospital, National Center for Cardiovascular Diseases, Chinese Academy of Medical Sciences and Peking Union Medical College, Beilishi Road No. 167, Xicheng District, Beijing 100037, China

A 69-year-old woman with a history of cerebral infarction and persistent non-valvular atrial fibrillation (AF) presented to the emergency room exhibiting inarticulate speech, urinary, and faecal incontinence. She had a cerebral infarction and AF in 2018 and had been taking dabigatran intermittently. One month after stopping using dabigatran, she experienced another cerebral infarction. Electrocardiography showed AF. Both transthoracic echocardiography (TTE) and transoesophageal ultrasound revealed a 67 × 60 mm giant mass occupying the majority of the left atrium with evidence of mitral valve orifice obstruction (*Figure 1A* and Supplementary material online, *Video S1*). Computed tomography (CT) of the left atrium showed a large, smooth, well-circumscribed filling defect in the enlarged left atrium, closely associated with the middle of the atrial septum. The mass was located close to the mitral valve orifice during the diastolic period (*Figure 1B* and Supplementary material online, *Video S2*). Based on both TTE and CT, a giant myxoma was suspected in the left atrium. The patient underwent surgical removal of the left atrial mass (*Figure 1C*). Gross pathological examination confirmed the mass was spherical with a smooth surface measuring 6.5 cm × 5.5 cm × 4.6 cm, which indicated a thrombus with no evidence of malignancy (*Figure 1D*).

**Figure 1:**
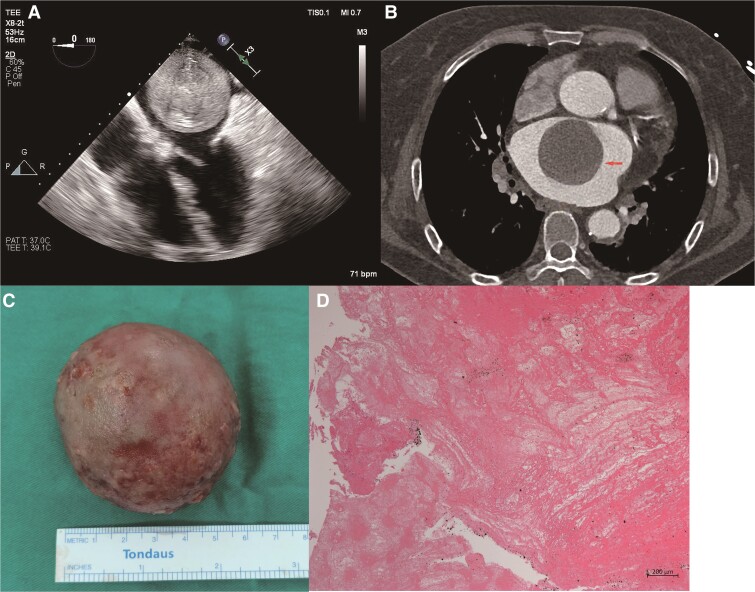
(*A*) Transoesophageal ultrasound detected a 58 × 55 mm moveable spherical mass in the left atrium. (*B*) Computed tomography showed a giant mass in the left atrium (the arrow shows the mass in the left atrium). (*C*) The giant left atrial mass is surgically removed. (*D*) Pathological examination confirmed the mass was a thrombus.

The patient had a difficult postoperative period complicated by lung infection and acute kidney failure. She died 2 weeks after surgery.

The differential diagnosis of a left atrial mass includes thrombus, vegetation, tumour, and other rare causes like septal aneurysm and septal haematoma.^1^ Ultrasound has no specificity in discriminating between thrombosis and myxoma. Cardiac magnetic resonance imaging has been proven to be a better diagnostic technique.^2^ While anticoagulation can lead to thrombus breakdown and subsequent embolism, appropriate management reduces this risk. Given the excessive space occupation in the left atrium and resultant mitral valve obstruction, surgical resection is often considered a more reasonable approach.

## Supplementary Material

ytae606_Supplementary_Data

## Supplementary material

**Consent:** The authors confirm that written consent for submission and publication of this case report, including images and associated text, has been obtained from the patient’s son, in line with COPE guidance.

**Funding:** None declared.

**Data availability:** The data underlying this article are available in the article and in its online Supplementary material.
